# Facilitating person-centered patient participation in kidney care—a process evaluation of a quasi-experimental study incorporating a tool and training of local implementation teams

**DOI:** 10.1186/s12913-024-11990-1

**Published:** 2024-12-12

**Authors:** Liselott Årestedt, Fredrik Uhlin, Ann Catrine Eldh

**Affiliations:** 1 Faculty of Health and Life Sciences, Department of Health and Caring Sciences, Linnaeus University, Kalmar/Växjö, 39182 Sweden; 2 Faculty of Medicine and Health Sciences, Department of Health, Medicine and Caring Sciences, Linköping University, Linköping, 581 83 Sweden; 3 Department of Nephrology, Region Östergötland, Linköping, 581 85 Sweden; 4 Department of Health Technologies, Tallinn University of Technology (TalTech), Tallinn, 19086 Estonia; 5 Department of Public Health and Caring Sciences, Uppsala University, Uppsala, Box 564, 751 22 Sweden

**Keywords:** Patient participation, Person-centered care, Process evaluation, Implementation, Facilitation, Context, Mixed methods

## Abstract

**Background:**

The transfer of innovations into healthcare is laden with challenges. Although healthcare professionals are expected to adopt and fulfil new policies, a more person-centered healthcare with conditions for preference-based patient participation is anticipated.

**Methods:**

The aim of the study was to evaluate two implementation strategies for person-centered patient participation in kidney care, including dissemination of a clinical toolkit, and additional training and support of internal facilitators. Nine Swedish kidney care units joined the study (August 2019–September 2021), strategically organized into: a control group (three sites, no support); a standard dissemination group (three sites, with a tool for patient participation and guidance disseminated to the site managers); and a facilitated implementation group (three sites, with the tool and guidance disseminated as above, plus a six-month support program for designated internal facilitators). This process evaluation was comprised of repeat interviews with managers (*n* = 10), internal facilitators (*n* = 5), recordings, and notes from the interventions, and Alberta Context Tool survey data (*n* = 78). Hybrid analyses comprised mixed methods: descriptive and comparative statistics, and qualitative descriptive analysis.

**Results:**

None of the control group sites addressed patient participation. While the standard dissemination sites’ managers received and appreciated the toolkit, they made no attempts to make further use of it. In the facilitated implementation group, five internal facilitators from three sites engaged in the support program. They welcomed the opportunity to learn about preference-based patient participation, and about implementation, including potentially enhanced opportunities for preference-based patient participation via the tool. Each site’s facilitators developed a separate strategy for the dissemination of the tool: the tool was used with a few patients in each site, and only some staff were involved. Although noting a general interest in improving patient participation, the internal facilitators described limited local support. Rather, they suggested a longer support program and more local backing and engagement.

**Conclusions:**

Facilitating person-centered patient participation is complex, given the need to address attitudes, beliefs, and behaviors. This study indicates slow uptake and change, and more efficient strategies are needed to ensure the fundamentals of care remain accessible to all.

**Supplementary Information:**

The online version contains supplementary material available at 10.1186/s12913-024-11990-1.

## Background

An increasing amount of scientific literature demonstrates that the transfer of innovations into healthcare practice is laden with challenges [1]. Even the implementation of high-quality evidence is associated with barriers, linked to the knowledge in question, the context into which it shall be translated, and the strategies employed to facilitate its progression into daily practice [2]. When it comes to policies, there is often even less evidence to begin with to promote change, but every so often there is a norm sustaining a program change; end-users, like healthcare professionals, are expected to adopt essentials of such policy as part of the implementation process [3]. While such adoption is key for implementation, it is estimated to occur as a result of a reasonable uptake of or agreement with proposed values. This seems to be the case with, for example, patient participation, which has been repeatedly suggested as an ideal standard in healthcare services [4]. Most professionals would likely be willing to agree that they promote optimal conditions for patients to engage in their care and health issues, although this is not always the case; rather, further efforts are required to understand what enables and hinders the implementation of person-centered opportunities for patient participation [5].

Patient participation has been on the agenda for at least 50 years, but healthcare professionals and organizations still do not fully embrace their responsibility to enable patients to engage in a way and to the extent they prefer [6]. Although healthcare professionals wish to engage patients, staff efforts are often based on their own assumptions regarding in what way and to what extent patients should participate [7, 8]. Patients, on the other hand, may—or may not—share the same assumptions. In kidney care, where patient engagement is suggested for a range of perspectives and activities, we found around 50% of patients receiving opportunities to engage in a way matching their preferences for participation [9] (with up to 20% of the patients expected to be more involved than they would prefer, or have fewer opportunities to be engaged than they prefer [9]), better opportunities to achieve more person-centered patient participation should be implemented.

## Methods

### Aim

The aim of this study was to evaluate two implementation strategies: the dissemination of a clinical tool, and additional training and support of internal facilitators (IF) for person-centered patient participation in kidney care.

### Design

The process evaluation was part of a hybrid study [10], including the effectiveness of the intervention reported elsewhere [11]. This study is reported considering Standards for Reporting Implementation Studies (StaRI) Statement [12].

### Setting and sample

Nine out of eleven kidney care sites in southeast Sweden agreed to participate in the study (two declined due to staffing issues). Seven of these sites had participated in a prior study, on staff and patient conceptualisation and experience of patient participation [7, 8]. The sites represented university, regional, and local hospitals; all sites had outpatient dialysis units, and seven of them also provided predialysis outpatient kidney care. At all sites, the majority of staff were nurses, but all sites also employed assistant nurses and physicians.

To ensure that the intervention and control groups were as equal as possible, sites were strategically organized into three groups with three sites each: a control group (CG), a standard dissemination group (SDG), and a facilitated implementation group (FIG) (the two latter representing intervention groups).

### Interventions

Recognizing that positive outcomes are dependent on strategies facilitating the implementation of a particular object in a particular context, the project was framed by the integrated Promoting Action on Research Implementation in Health Services program, i-PARIHS [13]. It tested two interventions manufactured on the following assumptions:

1. Swedish healthcare professionals have been compelled to provide for patient participation since at least 1982 (when the national healthcare act was introduced), and the introduction of a patient act (in 2015) and are likely aware of their obligation.
2. There is increasing awareness of the need for person-centered care, due to various efforts to improve opportunities for patients to engage in their health and healthcare issues [14].
3. Furthermore, many healthcare professionals and organizations are well-rehearsed in change management and have prior experience of addressing barriers while implementing knowledge or policies into practice [15].

Accordingly, this study established a control group (CG; three units) which received no prompts at all for enabling more person-centered patient participation.

To balance between providing enough support to facilitate knowledge implementation and taking advantage of the preparedness of staff and organization, empowering their potential to facilitate and sustain better and safer care [13], we set up two strategies to facilitate the implementation of person-centered patient participation in kidney care, in comparison with the CG and each other. Both strategies are further described with theoretical assumptions in Additional file 1: Table 1 [16, 17]. In short, the strategies represented:

- A standard dissemination group (SDG) of three sites to which a toolkit for patient participation was sent via e-mail at the start of the interventions (21 October 2019) to two managers per site: the first-line manager and the head of the unit.
- A facilitated implementation group (FIG). At the start of the interventions, that is 21 October, 2019, this group of three sites received the same dissemination toolkit as above (also sent to the first-line managers and the head of units). In addition, four months prior, the managers of the FIG (three sites) were asked to assign two healthcare professionals to act as internal facilitators, IFs [18], for patient participation. They were solicited to consider three characteristics when selecting their IFs: a) IFs should have an interest in developing and improving clinical healthcare; b) IFs managers and colleagues should trust them to be able to support change and improvements; and c) IFs should be willing to collaborate (with each other). By recruitment of the IFs, the FIG was offered a lean six-month intervention program, commenced the same date as the toolkit dissemination (October 21), ending 24 March 2020. In short, the FIG intervention was delivered as an initial two-day meeting, followed by scheduled monthly individual or group sessions via video conference, in accord with the IFs’ choice of format, for five consecutive months.

The interventions were developed and delivered by two external facilitators, EFs [13]: one implementation expert, also an expert in patient participation, plus a kidney care expert. Furthermore, an introduction lecture that one of the units had arranged (with one of the researchers on the team, per initiative of the management) for all staff prior to the beginning of the intervention, was offered to all IFs and their sites.

### Data collection

This process evaluation was composed of interviews with managers (Table 1) and interviews with internal facilitators (Table 2), data from the IF training and implementation support program, and data on the local organizational context from the Alberta Context Tool survey (n = 78, nurses only), collated from Aug 2019 through Sept 2021. An overview of the data that was collected and employed is presented in Table 3.

**Table 1:** Managers of partaking sites

Group	Manager (M) and Case (A–I)	Sex	Position	Interview date
**Control Group (CG)**	MD	Woman	Manager	10/03/19 04/21/20
	MG	Woman	Manager	10/02/19 04/16/20
	MH1	Woman	Deputy manager	10/08/19 04/14/19
	MH2	Woman	Deputy manager	09/30/19 04/27/20
**Standard Dissemination Group (SDG)**	ME / F	Woman	Manager	12/11/19 05/13/20
	MI	Woman	Manager	12/18/19 04/22/20
**Facilitated Intervention Group (FIG)**	MA	Woman	Manager	10/04/19 04/28/20
	MB	Woman	Manager	10/07/19 04/15/20
	MC1	Woman	Manager	10/04/19 05/06/20
	MC2	Woman	Deputy manager	12/19/19 04/29/20

**Table 2:** Internal Facilitator demographics

		**Sex**	**Profession**	**Type of Kidney care**	**Diary notes**	**No. of interviews**
Case A	IFA1	Woman	Nurse	Predialysis	x	2
	IFA2	Man	Nurse	Hemodialysis	x	2
Case B	IFB1	Woman	Nurse	Hemodialysis	-	2
	IF B2	Woman	Nurse	Hemodialysis	-	2
Case C	IFC	Woman	Nurse	Predialysis	x	2

**Table 3:** Data collected and employed for the process evaluation

Data source	Type of data	No. of participants	Amount of data
Interviews, managers	Verbatim transcripts, text	Ten (across nine sites)	129 pages (single space)
Interviews, internal facilitators (IFs)	Verbatim transcripts, text	Five (across three sites)	86 pages (single space)
The clinical intervention tool and its support for dissemination	4Ps	Distributed via e-mail to six managers and their head of unit (in the standard dissemination group (SDG) and the facilitated intervention group (FIG) sites, respectively	The 4Ps: two plus two pages The accompanying e-mail cover letter (1 page per type of site) User manual for the 4Ps (2 pages) 10 PPTs for dissemination
Training and support material for IFs in the facilitated intervention group (FIG)	PowerPoint slides plus description of the purpose and theoretical foundation of program components	Immaterial	43 pages
Notes from training and support sessions	Handwritten reflections of the researcher in charge of the training and support program	Immaterial	Eight pages
Recordings from all training and support sessions	Voice recordings	Immaterial	11 h and 19 min
IF diaries	Copies of handwritten or Word documents	Immaterial	28 pages
Alberta Context Tool	Survey	78 nurses (out of 98 responding staff) across 8 sites*	78 surveys

^*^Two sites were run by the same staff on rotation, with the same management

Individual interviews with managers and IF were conducted twice: manager interviews at the start and the end of the intervention period, and IFs at the end of intervention period and in a follow-up interview one year later. All participants received verbal and written information about the study, including information about the voluntary nature of their participation. In accordance with the World Medical Association’s ethical principles [19], [Accessed November 25, 2022] ethical approval was obtained by the Regional Ethical Review Board of Linkoping, Sweden (ID 2019–02748 and 2020–04296), and all interviews were performed following individual verbal and written informed consent.

The interviews were performed by the first author and a PhD candidate on the research team and followed semi-structured interview guides (based on a prior realist evaluation) [20]. To counteract bias, these researchers were blinded to the intervention and control groups at the time for management interviews. Consequently, no manager was asked specifically about their experience of any dissemination and/or implementation support program, though could voluntarily bring this up. Understandably, the later IF interviews (post-intervention × 2) were not blinded. The audio-recorded interviews with managers lasted 9–34 min and the interviews with IFs lasted 19–42 min. All interviews were then transcribed verbatim by an authorized secretarial service.

Data also included diary notes by the IFs (three out of five facilitators completed and submitted these notes), notes from the researchers performing the support program, copies of all material used and handed to the IFs, and voice recordings from all support program meetings of the implementation intervention.

Lastly, the Alberta Context Tool, ACT [21], was distributed at baseline (that is, prior to the interventions) to all nursing staff across sites via the managers, who were asked to share these via pigeon-hole mailboxes. ACT is a recognized survey previously translated (and validated) into Swedish [21], used for the measurement of eight concepts regarding organizational context in complex healthcare settings [22]. General reminders were sent to the managers, asking them to nudge staff to respond. Data were used to compare local contexts for the three groups (CG, SDG, and FIG). Furthermore, particular items were addressed to map context factors suggested to impact knowledge implementation and/or change: to what extent one’s manager a) attends to and acts to settle issues, b) actively supervises others in their work; and c) to what extent the team works to provide patients with what they need; and d) how often the team performs a care plan together with the patient and their next of kin.

### Analysis

For the transcribed interviews, a descriptive qualitative analysis was used [23, 24]. In addition, a mixed methods analysis, as defined by Sandelowski [25], was applied to all data, as described below.

Analyses began with the IF interviews. These were analyzed inductively, so as to understand their experiences of the support program, their role, and their context. Furthermore, the analyses addressed what they described as having performed and achieved as IFs.

Secondly, the IFs’ diary notes and material from the support program were analyzed (the latter by means of both training material and recorded sessions: a total of 11 h, including all sessions). This analysis phase employed a deductive approach, by means of the following steps, as suggested by Linnan & Steckler [26]: dose delivered (that is, what content was delivered to the IFs, in what format, by whom, when and the extent); dose received (what parts of the support program the IFs attended); and fidelity (what, if any, adjustments were made to the support program, when, and why).

Thirdly, the transcribed interviews with managers (*n* = 10) were added to the analysis. These texts were addressed with a descriptive qualitative approach (as above), identifying what managers described they had done, how, and why regarding patient participation at their sites during the project’s lifetime.

In addition, all data from the support program was analyzed, relating the ‘dose delivered’ and ‘dose received’ aspects described by IFs (and managers, if demonstrated) to what was manifested in the planned and performed training, material delivered, and the audio recordings. ACT data was organized into the three groups: CG, SDG, and FIG, as previously described, to investigate whether there were differences in organizational context between groups. For the analysis, non-parametric tests were used (Kruskal Wallis and Mann–Whitney U-tests) to compare each item; the level of significant variation between sites was set to *p* < 0.05.

In the fourth and final phase, a summary of all emerging outcomes was formed, inspired by a realist approach [27]:

1. What worked?
2. For whom?
3. In what context?
4. Why or why not?
5. With what outcomes?

## Results

The findings are presented in the following order: a report of the baseline context and the intervention delivery and refinements, followed by summaries of what worked (or did not work); how the interventions were perceived, and enacted (or not enacted) and why things worked (or did not work) and with what outcomes, respectively. Quotations to visualise the results (marked with which profession or position it represents, with code for site as presented in Tables 1 and 2).

### The kidney care context

Managers representing all nine sites considered patient participation important. They pronounced that patient participation was provided for in their sites, describing that patient participation is eminent in kidney care; it was considered addressed because of the long relations established with the patients with end-stage kidney disease.

*“We have our way of working with patient participation… Trying really to involve the patients. This is incorporated with all staff, although we have no particular means for this.”* (Manager MI).

Further, the managers described that in general, their staff should enable patient participation (but described neither how this was conceptualised nor how it was facilitated).

*“I thought it was very interesting ‘cause patient participation is a concept that we discuss. And it has turned out it means different things for different people. So, (…) I believe we have kept to our aim, for our patients to be as involved as possible in their care and treatment.”* (Manager MC2).

The analysis of the ACT showed no statically significant differences (Kruskal Wallis) in demographics between the three groups (CG, SDG and FIG) (that is: responding staffs’ gender, age, level of education, profession). Variables linked to employment also did not differ between the sites (years of experience, working hours, and type of employment). For the eight dimensions of organizational context (leadership, culture, evaluation, social capital, formal interactions, informal interactions, structural and electronic resources, and organizational slack in staffing, space, and time) a statistically significant difference (Mann–Whitney U-tests) between the groups emerged only for the latter dimension (organizational slack) in four items describing resources: the SDG group sites had higher reports on having enough staff to get the necessary work done at baseline (*p* = 0.037), but reported the lowest score for having a private space for staff exchanges than CG and FIG (*p* = 0.002). For the remaining two items where there was a difference between the groups—time to talk about the patient care plan (*p* = 0.010) and time to talk about new clinical knowledge (*p* = 0.047)—the FIG group sites had the lowest reports compared to the sites within the CG and SDG groups. An additional file shows the results of ACT in more detail [see Additional file 2].

### The facilitation (interventions)

The standard dissemination and the implementation support program were mainly delivered as planned. Firstly, the distribution of the toolkit to the managers was made according to plan. All managers in the SDG and the FIG described having read the included means for patient participation and the guidance to support its implementation that they had received via e-mail. No site (besides one in the FIG that had called for and arranged for a pre-intervention seminar for all staff with one of the external facilitators) made contact or requested further support, although such seminars were advertised as free and available.

In the FIG, one site assigned only one IF (rather than the suggested two). Other adjustments regarded the web-based support: following the face-to-face start of the implementation, the support program was offered per site or jointly. The first two meetings were held per site, followed by a joint meeting, as suggested by the program. In addition, the third meeting was proposed and performed as joint meeting. At this point, the IFs expressed that they preferred the two final meetings be joint (rather than per site, as proposed by the EFs).

Midway through the implementation support program, when the IFs asked for support on how to assemble and capture patient reports by means of the 4Ps tool, one of the EFs constructed and shared two means: a means to assess an individual’s reports over time, and/or one for assembling and assessing a number of patient reports at one time point.

The IFs described the implementation support program as rich in content. It had contributed to their increased understanding and opened up new perspectives. External facilitators were described as affirming, enthusiastic, and encouraging. The group meetings had become valuable for exchanging experiences regarding working methods, and contributed to new ideas, problem solving, and support.

*“I thought the support programme was very good; it helped me understand the 4Ps tool. And, I learned of the studies reinforcing it. And how it’s used, thoughts on how to apply it. So it was worthwhile, rather than just being handed the tool. If you understand more, you can explain to your peers how to use it… Making it easier to motivate.* (IFB1).

As all IFs came from the same context—i.e., kidney failure care—and accordingly, there was an understanding of each other's context. The arrangement of initial physical meetings and then digital meetings was described as positive.

### What worked (or did not work): how the interventions were perceived, and enacted (or not enacted)

The managers found the tool (delivered to the standard dissemination and implementation support sites) to be appropriate and interesting. None of the managers commented on the support and guidance that accompanied the tool. Furthermore, no managers made any additional efforts to address patient participation in their site. Neither did they describe any additional efforts to address patient participation beyond the point of reading the e-mail with the toolkit for how to promote person-centered patient participation. Rather, the managers suggested that since their staff already provided for patient participation, they had no reason to suggest supplements; if the 4Ps tool was to be employed, it would either confirm current practice or could potentially lend a more accurate representation of the patients’ perspective on patient participation, according to the managers. For any further activity, the managers in the FIG suggested that they had appointed the IFs for such a commission.

For the IFs of the implementation support sites, there were opportunities to work with the implementation at each site between program meetings. The IFs had focused on the tool suggested in the intervention, the 4Ps, which was perceived as an aid to promote patient participation, by means of aiding the dialogue between patients and staff. The three sites’ IFs addressed implementation in different ways, as illustrated in Table 4.

**Table 4:** FIG site activities, described by internal facilitators

Case	Activities	IFs’ reflections
A	• Regular reconciliations between the IFs • Informed managers and other colleagues; used the PowerPoint presentation provided • Involved other nurses early in the process • Started on a small scale and then expanded with more nurses • Adapted information to included nurses • Had follow-up meetings • Tried different approaches to involve patients • Estimated time spent: a couple of hours a week	• Did not experience any problems to reach out with information • Managed to stick with the original idea and plan • Things turned out as planned but with fewer patients than anticipated • Lack of control over which patients were included • A fun and exciting project
B	• Did not prioritize the project • Talked about patient participation at a workplace meeting • Estimated time spent: short discussions, a few minutes, between the two IFs	• Wish they have done more activities and spent more time on the implementation • Clearer leadership would have been needed to succeed
C	• Worked on the intervention singlehandedly • Informed colleagues about the project • Estimated time spent: a couple of hours each week, alongside regular nursing tasks	• Difficulties reaching out to and including other staff • Lack of time and staff shortages at the unit • Alone as IF, missing a partner • IF’s expectations and ambitions not matched by peers or managers • Developed own ability for self-reflection

In one site, the IFs made a plan for the implementation of the 4Ps tool, starting with involving a few colleagues while notifying their managers and all fellow staff about the plan. After this, they included some patients to complete the 4Ps tool, although not as many as they initially planned to include. They described having support from their managers, who emphasized the importance of all staff to work in accordance with IF plans and to coordinate their efforts toward the same goal.

In two sites there were no strategic plans procured for the implementation; the IFs worked with implementation, primarily of the 4Ps tool, but described it as spontaneous.

### Why things worked (or did not work) and with what outcomes

The managers of the SDG and FIG sites referred to already-established working methods for person-centered care, engaging patients in their care. However, they also reflected a lack of a common strategy and approach to patient participation. The toolkit dissemination did not bridge any barriers to address these issues.

For the implementation support sites, FIG, the data (that is, recordings and external facilitators’ notes) claimed that the IFs turned to the tool and its implementation, rather than to the more general issue of patient participation like person-centered opportunities to engage in one’s health and care issues as a patient. Moreover, the external facilitators found the joint meetings of the program to initially aid local progress, when the IFs shared what they had done and what had worked. However, over time, the external facilitators identified that the IFs increasingly compared themselves to each other, imparting any status quo by claiming why they had not made any similar attempts or taken actions.

The IFs described difficulties in implementing the ideas they had from the program; they suggested that they would have needed to spend more time and energy to make it work.

*”You realise that there’s a lot more to do, to implement things. And perhaps, it requires more of persistent work… with repeated information… and that you keep at a certain issue, being more stubborn on what’s to be done. And to encourage and such… one's own attitude is very important then.”* (IFA2*).*

In addition, the IFs proposed that the intervention support program was too short; organizational factors such as too many patients, a high workload, colleagues on sick leave and/or vacation, and staff shortages were considered to hamper any implementation—in this case their implementation of the tool to address person-centered patient participation. Other parallel quality projects also made it difficult to engage colleagues in their efforts to make the implementation of patient participation work in practice.

Furthermore, once the intervention came to an end the COVID-19 pandemic created a further shortage of staff, making it harder for the IFs to advance and include additional patients as intended. Rather, at this point, staff were reallocated to other units, leaving kidney care with resources for only the most fundamental care, and patients’ face-to-face visits were cancelled whenever possible (protecting both the individuals from risks and enabling staff relocation).

Overall, the IFs described their role as facilitators of the patient participation implementation as more complex than they first imagined. They appreciated the learning opportunity and acknowledged that they had learned a lot from the support program (about both patient participation and knowledge implementation). If asked again, they would accept such an IF assignment. They described that they had learned more about their own attitudes and behaviors when engaging in direct patient encounters while providing kidney care. This enabled them to focus more on the patient's agenda, which was considered a new and different way of thinking, ensuring greater flexibility for their patients. This was described as difficult to incite in others but was considered more of an individual trajectory.

Despite their hardships, none of the IFs had asked for more support from their managers. Rather, noticing how their own ideas of patient participation and the opportunities they identified to improve the standard of care in their sites had altered—including addressing patients with regard to their preferences for and experiences of participating, they suggested that changing the norms of their workplace would require more time (rather than the six months that the intervention support program provided), and more people engaged in the process.

## Discussion

Recognising the patient voice and choice has been described as important for both patients and staff in the kidney care context [28]. While managers in this study described the kidney care context as commonly associated with patient participation, the conceptualization of patient participation was previously found to vary [8]: not only did patients and staff have different priorities [29], but patients suggested that their engagement varied, over time and with respect to their condition [7, 30]. This calls for further dialogue in healthcare encounters regarding enhanced person-centeredness in kidney care [31]; this project aimed to facilitate such amendments, with increased awareness, and means to sustain dialogue. The findings indicate an unconvincing impact of the dissemination strategy, and some (albeit slow and limited) influence with added support to internal facilitators, IFs.

Prior to the intervention, roughly 50% of kidney care patients were found to have had opportunities for participation matching their preferences; while almost every second patient did not, every fifth patient had a complete mismatch between their preferences for and experiences of participation [9]. During and following the intervention, there was no evident effect of it, but even with more preference-based patient participation, up to 12% of the patients were not sufficed with conditions for engagement matching their needs and resources; most often, this represented a preference to be more involved in their health and healthcare matters than facilitated [11]. This paper signifies that while both managers and the IFs appreciated the potential and actual support of the study, there were limited actions taken beyond those pertaining to a few individual patients, and some staff. We suggest this is primarily due to the innovation–context interplay [32] (but will return to the facilitation strategies later):

A. Regardless of the often frequent contact and long relationships established between patients and staff, particularly in dialysis care, encounters in kidney care are charged with the many technical aspects of lifesaving treatments. To have safe and effective procedures, processes have been streamlined, sometimes failing to recognize the more person-centered aspects of the people who are patients in kidney care [33, 34].
B. Patient participation is presumably a comprehensive concept, designating a humanistic stance for more person-centered care [35], yet is potentially challenging to apprehend and convey in everyday encounters without support [36].

Hence, it is reasonable that while both interventions aimed for a more general implementation of improved conditions for preference-based patient participation, the significance of considering the concept of patient participation was lost. A divergent conceptualisation of patient participation between staff and patients lingered [37], mainly steered by the staffs’ idea of how to promote engagement in health and healthcare. Yet, much like previous implementation efforts, the internal facilitators focused the tool rather than emphasising the potential need for more personcenteredness [38]. In hindsight, an alternative would have been to facilitate a patient-mediated intervention [39], recognising patients’ resources for learning of their participation and sharing of their lived experience [40]. The potential of addressing patient needs merit further investigation, although the fatigue that often occurs alongside severe chronic kidney failure [41] may exclude this option for patients undergoing hemodialysis (but may work in self-administered dialysis or outpatient care when in predialysis).

The focus on the patient participation tool can also be seen in view of the implementation fidelity; i.e., the degree to which the intervention was adopted and implemented as intended [42]. The IFs appreciated the support program, and while they described having altered their attitudes and engagement with their patients, they neither had nor attracted the resources required to facilitate a corresponding adoption among their peers. Rather, both managers (in SDG and FIG) as well as IFs (FIG) emphasized time as a crucial factor—here, a barrier—to addressing more person-centered patient participation. Consequently, the focus of the IFs and managers can be the operative mode connotated by the kidney care context, or simply a lack of time and energy to address a more major change in attitudes and routines. Healthcare management means engaging staff to craft a culture confident in making the necessary changes for the benefit of better and safer care [43]. Across these nine sites, the managers trusted staff to already facilitate patient participation, but the IFs described it as hard to ensure that enough time was set aside to work on the implementation of more person-centered patient participation. This is consistent with findings showing that time and work pressure are common barriers to enhancing patient participation, and nurses tend to focus on their tasks rather than integrating patients’ needs [44]. Another limitation highlighted by the IFs was staff shortage, making staffing a priority. This can procure an attitude among staff that their abilities to affect and change routines are limited, which in turn can prevent their involvement in future implementation of new innovations. A task-oriented management approach is known to hamper knowledge implementation [43], particularly when it comes to changes to nursing practice in favor of more person-centered care [44]. This would need to be further addressed in future projects and events.

Despite their hardships, the IFs grappled with the intervention on their own. The managers, on the other hand, expressed both that patient participation was already procured and that there was a lack of a common strategy and approach in terms of patient participation at their units. This indicates a need for a stronger relationship between managers and facilitators in knowledge implementation [43, 45]. This has lately been highlighted as a need to address both first-line managers, and upper-level management to facilitate implementation [46]. Promoting a fair context and scaffolding a person-centered context, managers’ attitudes to change need to focus on improvement processes, with first line managers acting as role models if and when new methods and evidence are introduced [47].

The facilitator role is complex, requiring knowledge both about implementation and the innovation in particular [48]. Efforts to facilitate IFs in this context and/or with the intention to improve person-centered conditions for patient participation would presumably benefit from a more detailed mapping of their barriers. This would include attitudes about the innovation and contextual factors, boosting IFs’ ability to tailor plans to bridge obstacles, and to envisage what enabling factors are available and/or can be accessed [42]. Changing staff attitudes towards more person-centered patient participation opportunities will likely take time and require further joint efforts, requiring a supportive healthcare environment for both patients and staff, and the adoption of change-oriented leadership [43, 47]. Our findings reinforce that IFs should serve in pairs or in teams, collaborating with management [43]; managers are vital in balancing external requirements with internal processes, and in encouraging and implementing new ideas [49]. While the kidney care context remains focused on patients’ opportunities for learning about their condition and treatment and providing such options [50], we suggest additional recognition of patients’ resources and preferences for being engaged would further meet calls for more person-centered care [51].

### Limitations

In spite of the intervention data on the planned and procured dissemination and support program, in addition to the IFs’ diaries and the interviews, details on how the IFs adopted and proceeded with the implementation in everyday working life were missing; the IFs diary notes were scarce (and described mostly practical activities), picturing a task-oriented approach. Though three out of the five IFs completed their diaries, any generalization of their plans is to be carefully interpreted. For context, we used both the Alberta Context Tool survey, and interviews across all sites; while this provided a picture of important contextual factors [52], such as available resources, there were negligible differences in organizational context according to results from the ACT analysis, making the three groups comparable. Further, any previous engagement in focus group discussions to explore the conceptualisation of patient participation [7] did not transpire in the current interviews. Since the interviews with the managers were guided by queries at a general level, further details about the implementation process may have been lost. On the other hand, this approach warranted less bias risk at that point. Still, a deeper understanding of both the contextual factors and the reach of the intervention(s) would have been facilitated by further staff interviews, illuminating if they had been addressed by their IFs and/or managers, and what worked in terms of the interventions. Any further evaluations of a refined implementation strategy to facilitate preference-based patient participation should include details grappling the costs of the intervention relative to outcomes [53].

## Conclusions

Knowledge implementation is acknowledged as complex, even if the innovation to be implemented is well-known and appreciated. In this study, both managers and IFs had a positive attitude regarding patient participation, but the IFs described limited readiness for preference-based patient participation. While all sites' managers suggested that patient participation is innate in the kidney care context, a perceived lack of time and a lack of relevant mandates hampered both managers and IFs when it came to facilitating more person-centered conditions. The IFs partaking in the support program increased their knowledge and understanding, which helped them reconsider how they enabled patient participation. This indicates a slow uptake and change, with benefits for some patients. More efficient strategies are needed to ensure patient engagement as a fundamental of quality of care is accessible for all.

## Supplementary Information

Additional file 1. Table 1. Overview of the intervention components, delivered to the SDG and FIG sites, respectively.

Additional file 2. Comparisons at group level for each ACT item/variable.

Additional file 3. Interview guide- Internal facilitators.

Additional file 4. Interview guide Managers.

Additional file 5. StaRI-checklist-for-author-completion.

## Data Availability

The datasets used and/or analyzed during the current study available from the corresponding author on reasonable request.

## Abbreviations

CG: Control Group
FIG: Facilitated Implementation Group
IF: Internal Facilitator
SDG: Standard Dissemination Group
