# Supplementary material for: Facilitating person-centered patient participation in kidney care—a process evaluation of a quasi-experimental study incorporating a tool and training of local implementation teams
Source: BMC Health Serv Res. 2024 Dec 12;24:1559. doi: 10.1186/s12913-024-11990-1 (PMC11636029; doi:10.1186/s12913-024-11990-1)
Supplement: Supplementary file 1 — Additional file 1. Table 1. Overview of the intervention components, delivered to the SDG and FIG sites, respectively. [file 12913_2024_11990_MOESM1_ESM.docx]

Additional file 1: Table 1. Overview of the intervention components, delivered to the SDG and FIG sites.

| Type of support | Date | Content | Purpose and theoretical assumptions | Where | Who |
| --- | --- | --- | --- | --- | --- |
| Dissemination of knowledge transfer toolkit | 21 October, 2019 | 1. A tool to facilitate a shared understanding of the patients’ preferences for, and experiences of, participation: - the Patient Preferences for Patient Participation tool, the 4Ps [1].  2. A two-page information leaflet, providing the background on the tool and information on how to facilitate its use in clinical practice, including a follow-up and assessment of whether patients had had opportunities to engage in their care in accordance with their preferences.  3. A PowerPoint presentation including both current knowledge about patient participation, and guidance to implementing the 4Ps to facilitate preference-based patient participation.  4. The cover letter outlined the content and how this could be used with local healthcare professionals. | It was suggested that this toolkit should be used to augment patient participation as an element of quality of care. The dissemination strategy aligned with national and regional authorities’ communication of information to target groups [2, 3], in this case on patient participation in healthcare organisations. | Sent via a joint e-mail to two managers per site. | The first-line manager and the head of the unit at each of the SDG and FIG sites. |
| Workshop | 21 October, 2019 | 1. Patient participation, in general, 2. patient participation in kidney care, 3. knowledge implementation, and 4. experiences of improvement and change.   For each of the sessions, a PowerPoint was presented to which the IFs had access both during and after the intervention. In response to the IFs queries, evidence and tools were assembled and shared throughout the intervention. | Introduction and seminars; All interactions were based on dialogue, addressing the needs and plans of the IFs, and their perceived local contexts. It assembled the renowned integrated Promoting Action on Research Implementation in Health Services framework [4]: aiming for the further dissemination of preference-based patient participation strategies, it encouraged the IFs to employ their previous knowledge and experience of quality improvement, yet with candid sessions addressing how to identify and bridge barriers to change in clinical practice. Facilitating facilitators to facilitate [4, 5], the strategy incorporated a problem-based learning component [6], addressing what knowledge and experience the IFs had, yet expanding their skills for the benefit of themselves and their peers. | Conference center | FIG: Joint, all IFs plus the two external facilitators |
| Workshop | 22 October, 2019 | Making an implementation plan  Capturing and evaluating processes and outcomes  Further intervention support  Recap and reflections | Planning and procuring plans for implementation; a knowledge-to-action approach was suggested to benefit the IFs prior improvement experience, scaffolding their knowledge-broker role [7, 8]. | Conference center | FIG: Joint, all IFs plus the two external facilitators |
| Tele-conference | 19, 21 and 25 November, 2019 | What has been done, how, for whom, and with what outcomes | Reflections on barriers and enablers, plan forward; a knowledge-to-action approach was suggested to benefit the IFs prior improvement experience, scaffolding their knowledge-broker role [7, 8]. | ZOOM | FIG: Per site (IFA2 absent) |
| Tele-conference | 19 December, 2019 | What has been done, how, for whom, and with what outcomes | Reflections on barriers and enablers, plan forward; a knowledge-to-action approach was suggested to benefit the IFs prior improvement experience, scaffolding their knowledge-broker role [7, 8]. | ZOOM | FIG: Per site (IFA1 absent) |
| Tele-conference | 21 January, 2020 | What’s working, why or why not | Sharing experiences, plan ahead, as above. | ZOOM | FIG: Joint (IFA1 absent) |
| Tele-conference | 20 February, 2020 | A further understanding of patient participation | Tackling barriers among fellow staff and patients, as above. | ZOOM | FIG: Joint (IFB2 absent) |
| Tele-conference | 24 March, 2020 | What have we learned and where do we go from here | Experiences of change management and learning opportunities; a knowledge-to-action approach was suggested to benefit the IFs prior improvement experience, scaffolding their knowledge-broker role [7, 8]. | ZOOM | FIG: All |

References used in additional file 1:

1. Eldh AC, Luhr K, Ehnfors M. The development and initial validation of a clinical tool for patients' preferences on patient participation—The 4Ps. Health Expect. 2015;18(6):2522–35.
2. Rogers EM. Diffusion of innovations. 5th ed. New York: Free Press; 2003.
3. Grol R, Wensing M, Eccles M. Improving patient care. The implementation of change in clinical practice. Elsevier, 2005.
4. Harvey G, Kitson A. PARIHS revisited: from heuristic to integrated framework for the successful implementation of knowledge into practice. Implement Sci. 2016;11:33.
5. Eldh AC, Halleberg Nyman M, Joelsson-Alm E, Wallin L. Facilitating facilitators to facilitate—Some general comments on a strategy for knowledge implementation in health services. Frontiers in Health Services, Implement Sci. 2023;3.
6. Bhogal SK, Murray MA, McLeod KM, Bergen A, Bath B, Menon A, Kho ME, Stacey D. Using problem-based case studies to learn about knowledge translation interventions: an inside perspective. J Contin Educ Health Prof. 2011 Fall;31(4):268-75.
7. Field B, Booth A, Ilott I, Gerrish K. Using the Knowledge to Action Framework in practice: a citation analysis and systematic review. Implement Sci. 2014 23;9:172.
8. Kitson AL, Harvey G. Methods to Succeed in Effective Knowledge Translation in Clinical Practice. J Nurs Scholarsh. 2016;48(3):294-302.
