# Supplementary material for: Facilitating person-centered patient participation in kidney care—a process evaluation of a quasi-experimental study incorporating a tool and training of local implementation teams
Source: BMC Health Serv Res. 2024 Dec 12;24:1559. doi: 10.1186/s12913-024-11990-1 (PMC11636029; doi:10.1186/s12913-024-11990-1)
Supplement: Supplementary file 2 — Additional file 2. Comparisons at group level for each ACT item/variable. [file 12913_2024_11990_MOESM2_ESM.docx]

**Additional file 2.**

**Comparisons at group level for each ACT item/variable**

|  | **Variable No.**  **(ACT)** | **Control Group**  **(CG). n=29** | | **Standard Dissemination Group (SDG). n=14** | | **Facilitated Implementation Group (FIG). n=55** | | **P-value**  **(Kruskal Wallis)** |
| --- | --- | --- | --- | --- | --- | --- | --- | --- |
|  |  | Mean | ± SD | Mean | ± SD | Mean | ± SD |  |
| ***LEADERSHIP*** | 01 | 3.24 | 1.25 | 3.64 | 0.89 | 3.33 | 1.20 | .671 |
|  | 02 | 3.66 | 1.03 | 3.64 | 0.89 | 3.73 | 1.05 | .966 |
|  | 03 | 3.76 | 0.97 | 4.07 | 0.80 | 3.71 | 1.30 | .757 |
|  | 04 | 3.55 | 1.25 | 3.86 | 0.64 | 3.67 | 1.11 | .934 |
|  | 05 | 3.52 | 1.16 | 3.64 | 0.72 | 3.67 | 0.92 | .925 |
|  | 06 | 3.10 | 1.16 | 3.00 | 0.93 | 3.24 | 1.22 | .792 |
| ***CULTURE*** | 07 | 3.97 | 0.76 | 4.29 | 0.45 | 3.91 | 0.61 | .162 |
|  | 08 | 4.48 | 0.56 | 4.50 | 0.63 | 4.25 | 0.72 | .276 |
|  | 09 | 4.00 | 0.53 | 4.00 | 0.76 | 3.75 | 0.67 | .199 |
|  | 10 | 3.79 | 0.92 | 3.93 | 0.80 | 3.82 | 0.79 | .879 |
|  | 11 | 4.48 | 0.56 | 4.50 | 0.63 | 4.49 | 0.57 | .978 |
|  | 12 | 4.31 | 0.53 | 4.36 | 0.48 | 4.33 | 0.57 | .972 |
| ***FEEDBACK (evaluation)*** | 13 | 3.93 | 0.94 | 3.36 | 1.04 | 3.40 | 0.95 | .201 |
|  | 14 | 3.93 | 0.69 | 3.36 | 1.04 | 3.49 | 1.01 | .097 |
|  | 15 | 3.83 | 0.70 | 3.14 | 1.06 | 3.60 | 0.98 | .130 |
|  | 16 | 3.48 | 0.77 | 3.00 | 1.20 | 3.31 | 0.95 | .485 |
|  | 17 | 3.48 | 0.62 | 3.14 | 1.06 | 3.33 | 0.81 | .610 |
|  | 18 | 3.07 | 0.87 | 3.14 | 1.25 | 2.84 | 0.91 | .426 |
| ***FORMAL INTERCATIONS*** | 19 | 0.47 | 0.43 | 0.50 | 0.42 | 0.34 | 0.46 | .228 |
|  | 20 | 0.12 | 0.28 | 0.32 | 0.41 | 0.12 | 0.30 | .062 |
|  | 21 | 0.02 | 0.09 | 0.04 | 0.13 | 0.01 | 0.07 | .584 |
|  | 22 | 0.17 | 0.33 | 0.29 | 0.36 | 0.12 | 0.25 | .188 |
| ***INFORMAL INTERACTIONS*** | 23 | 0.95 | 0.20 | 0.86 | 0.29 | 0.82 | 0.36 | .206 |
|  | 24 | 0.74 | 0.39 | 0.75 | 0.37 | 0.75 | 0.40 | .979 |
|  | 25 | 0.34 | 0.37 | 0.29 | 0.36 | 0.43 | 0.43 | .493 |
|  | 26 | 0.17 | 0.33 | 0.14 | 0.29 | 0.09 | 0.25 | .394 |
|  | 27 | 0.19 | 0.36 | 0.04 | 0.13 | 0.10 | 0.28 | .241 |
|  | 28 | 0.09 | 0.23 | 0 | 0 | 0.05 | 0.21 | .297 |
|  | 29 | 0.14 | 0.32 | 0.07 | 0.17 | 0.08 | 0.21 | .882 |
|  | 30 | 0.66 | 0.42 | 0.68 | 0.31 | 0.73 | 0.39 | .603 |
|  | 31 | 0.45 | 0.48 | 0.32 | 0.36 | 0.30 | 0.39 | .442 |
| ***CONNECTIONS AMONG PEOPLE (social capital)*** | 32 | 4.59 | 0.62 | 4.64 | 0.48 | 4.35 | 0.51 | .085 |
|  | 33 | 4.52 | 0.62 | 4.57 | 0.62 | 4.45 | 0.53 | .659 |
|  | 34 | 4.00 | 0.83 | 4.29 | 0.80 | 3.93 | 0.83 | .210 |
|  | 35 | 4.66 | 0.54 | 4.57 | 0.82 | 4.53 | 0.60 | .681 |
|  | 36 | 4.45 | 0.62 | 4.50 | 0.73 | 4.38 | 0.62 | .649 |
|  | 37 | 4.07 | 0.91 | 4.29 | 0.59 | 4.00 | 0.76 | .370 |
| ***STRUCTURAL AND ELECTRONIC RESOURCES*** | 38 | 0.12 | 0.25 | 0.11 | 0.28 | 0.04 | 0.13 | .191 |
|  | 39 | 0.21 | 0.31 | 0.18 | 0.31 | 0.12 | 0.25 | .338 |
|  | 40 | 0.50 | 0.44 | 0.25 | 0.37 | 0.42 | 0.41 | .194 |
|  | 41 | 0.57 | 0.41 | 0.43 | 0.42 | 0.35 | 0.38 | .063 |
|  | 42 | 0.74 | 0.31 | 0.64 | 0.40 | 0.80 | 0.28 | .376 |
|  | 43 | 0.64 | 0.32 | 0.61 | 0.34 | 0.68 | 0.35 | .641 |
|  | 44 | 0.43 | 0.36 | 0.43 | 0.32 | 0.33 | 0.36 | .330 |
|  | 45 | 0.43 | 0.43 | 0.36 | 0.40 | 0.30 | 0.42 | .342 |
|  | 46 | 0.29 | 0.38 | 0.14 | 0.29 | 0.38 | 0.42 | .118 |
|  | 47 | 0.74 | 0.34 | 0.75 | 0.37 | 0.56 | 0.39 | .068 |
| ***ORGANIZATIONAL SLACK*** | 48 | 4.07 | 0.74 | 4.43 | 0.62 | 3.75 | 1.24 | **.037** |
|  | 49 | 3.86 | 0.82 | 4.07 | 0.70 | 3.75 | 1.16 | .550 |
|  | 50 | 4.21 | 0.76 | 2.86 | 1.06 | 3.84 | 1.04 | **.002** |
|  | 51 | 4.21 | 0.85 | 3.71 | 1.39 | 4.13 | 1.21 | .497 |
|  | 52 | 4.14 | 1.31 | 4.36 | 1.29 | 3.93 | 1.20 | .446 |
|  | 53 | 3.45 | 0.67 | 3.50 | 0.73 | 3.49 | 0.71 | .982 |
|  | 54 | 3.66 | 0.92 | 3.71 | 0.59 | 2.98 | 0.98 | **.010** |
|  | 55 | 3.34 | 0.71 | 3.50 | 0.63 | 2.98 | 0.86 | **.047** |

Variable No. (ACT) correspond to every item in the Alberta Context Tool survey. Mean ± SD is values from the score of the item.
