# Supplementary material for: Facilitating person-centered patient participation in kidney care—a process evaluation of a quasi-experimental study incorporating a tool and training of local implementation teams
Source: BMC Health Serv Res. 2024 Dec 12;24:1559. doi: 10.1186/s12913-024-11990-1 (PMC11636029; doi:10.1186/s12913-024-11990-1)
Supplement: Supplementary file 3 — Additional file 3. Interview guide- Internal facilitators. [file 12913_2024_11990_MOESM3_ESM.docx]

**Interview guide, internal facilitator**

Please tell me about your thoughts:

- when you first heard about the project?

- prior to the intervention program?

What is your experience of the program? Is there anything in particular that you recall?

Please consider the value of the intervention program:

- in relation to your role as a facilitator?

- in your usual (professional) role in the unit?

How did you experience the support? Is there anything in particular that you remember?

Please consider the virtual meetings of the intervention program - how did you use these?

Please tell me about your role as a facilitator…

If you think about what came up on the tutorial(s) – how do you use it in your work?

What has been in focus in your unit?

How were the completed activities in relation to what you planned? Who has been involved besides you (and your facilitator colleague, if appropriate)?

Can you estimate the amount of time spent on the project? (If difficult, e.g. how many hours per week do you spend on the activities described.)

What has affected your ability to carry out the work and the activities associated with the project? (Probes: How, why, and in what situations?)

What was the significance of your activities? Was that the impact you intended?

How would you describe the support from employees and managers in your role and work as facilitator?

In retrospect - what would you have done differently?

Is there anything that has been difficult? In such case: what – please exemplify? What did you do to circumvent/overcome any difficulty/difficulties?

What has gone well?

Would you consider taking on a similar assignment?

What would you consider before taking on such a role again?

What advice would you give to someone who was to take on a similar role?

This guide was developed for the Patient Participation in Kidney Care study, by

Eldh et al, based on Rycroft-Malone et al (2018) 2019-03-08
