# Supplementary material for: Facilitating person-centered patient participation in kidney care—a process evaluation of a quasi-experimental study incorporating a tool and training of local implementation teams
Source: BMC Health Serv Res. 2024 Dec 12;24:1559. doi: 10.1186/s12913-024-11990-1 (PMC11636029; doi:10.1186/s12913-024-11990-1)
Supplement: Supplementary file 4 — Additional file 4. Interview guide Managers. [file 12913_2024_11990_MOESM4_ESM.docx]

**Interview guide, manager**

As you may recall from the written information we have sent, patient participation can mean different things to different people at different times. For some, it can also vary depending on what type of healthcare they are in contact with, or what transition they make. Since there is no right or wrong, I would like to start by asking you to tell me what you think of when you think of patient participation? (Probes, if needed: think of a situation when you considered a patient participating… Or, if needed: a situation when you sensed that a patient was not participating…)

Please tell me what you think enables patient participation? (Probes, if needed: how it is now… How it was in the past…)

If you think about your dialysis unit – what affects patient participation? (Probes: factors such as the kidney disease? Symptoms? The premises? How the care is organised? The staff who work in the unit? The teamwork?)

If you were to change anything in terms of patient participation, what would it be?

When you think of, and talk about, patient participation – is there anything in particular that comes to mind?

This guide was developed for the Patient Participation in Kidney Care study, by

Eldh et al 2019-03-08
